# Innate versus adaptive immunity in kidney immunopathology

**DOI:** 10.1186/1471-2369-14-138

**Published:** 2013-07-08

**Authors:** Hans-Joachim Anders

**Affiliations:** 1 Renal Division, Medizinische Klinik und Poliklinik IV, Klinikum der Universität München, Ziemssenstr. 1, D-80336, Munich, Germany

**Keywords:** Glomerulonephritis, Acute kidney injury, Chronic kidney injury, Chemokines, Macrophages, B cells, T cells

## Abstract

Most kidney disorders involve some degree of inflammation, i.e. induction of pro-inflammatory mediators and leukocyte recruitment. But what are the factors that determine inflammation as a trigger or a consequence of kidney injury? Which types of renal inflammation can be targeted by the novel more selective immunosuppressive and anti-inflammatory agents? How to dissect the mechanisms behind innate and adaptive immune responses that are orchestrated inside or outside the kidney but both cause renal immunopathology i.e. renal inflammation? How to dissect leukocytic cell infiltrates into pro-inflammatory leukocytes from anti-inflammatory and pro-regenerative leukocytes? How to dissect leukocytes that support epithelial repair from those that promote renal fibrosis. The term ‘renal inflammation’ has moved far beyond the descriptive category of ‘mixed leukocytic cell infiltrates’ as commonly described in kidney biopsies. It is time to face the complexity of renal inflammation to finally benefit from the new age of novel immunomodulatory medicines.

## 

Inflammation is one of several danger response programs that helps to control dangers that disrupt kidney homeostasis [1,2]. Historically, inflammation was clinically defined by the combination of *rubor, calor, dolor, tumor*, and *functio laesa*. In the 18th and 19th century the concept of cellular pathology evolved and since then leukocytic cell infiltrates were taken as a hallmark of what we call tissue inflammation. Until today the presence of increased numbers of leukocytes in body fluids such as blood, liquor, urine or within joints or the pleural and peritoneal cavity is taken as a biomarker of inflammation. Based on the work of Jenner, Behring, Kitasato, Ehrlich, and Lahnsteiner immunological memory was discovered and first thought to solely depend on humoral factors, the immunoglobulins [3]. This view was subsequently refined by the discovery of the T and B lymphocytes and later by the identification and characterization of antigen-presenting cells [3]. In the 20th century research in the field of immunology was dominated by the growing understanding of the complexity of antigen-specific ‘adaptive’ immunity, immune tolerance, and immune memory, a field much stimulated by the implementation of organ transplantation into clinical practice [3].

Despite seminal advances on kidney physiology and pathology in the 19th century nephrology as a distinct medical discipline did not evolve before the second part of the 20th century, largely driven by technical advances in renal replacement therapies [4,5]. The growing understanding of renal inflammation as a pathway to chronic kidney disease was dominated by the pathophysiological concepts of immune complex disease, antigen-specific immunity in interstitial nephritis, and alloimmunity of the kidney allograft [6]. It was not before the discovery of the complement system, the Fc receptors, and the cytokines that innate immunity became a central topic of interest [6]. Chemokines raised further attention on the mechanisms of neutrophil and macrophage recruitment, which, obviously, contributed to immunopathology of the kidney in an antigen-independent manner [7].

However, upon the discovery of the Toll-like receptors [8], the RIG-like helicases [9], and the inflammasomes [10] in the 1990s the scientific community shifted its full attention towards the innate immune system [11]. The innate and the adaptive immune system are different from many points of view as listed in Table 1. The evolution of the innate immune system dates back to the first single cell organisms, while adaptive immunity evolved not before the appearance of the jawed cartilaginous fish around 500 million years ago [12]. Since then evolution has imprinted a tight connection between innate and adaptive immune reponses for the sake of most efficient pathogen control [13]. But what is the impact of the evolving understanding of innate immunity on renal science?

**Table 1:** Differences in innate and adaptive immunity

	**Innate immunity**	**Adaptive immunity**
Recognition receptors (R)	Complement-R	B cell receptors
	Mannose-R	T cell receptors
	Toll-like-R	MHC I
	Inflammasomes	MHCII
	Low affinity immunoglobulins	High affinity IgG
Receptor clonality	Non-clonal	clonal
Receptor genes	Single gene, no rearrangement required	Encoded in gene segments Rearrangement required
Receptor agonists	Molecular patterns	Antigenic epitopes
Time delay of response	Immediate	Delayed
Effector mechanism	Opsonization, phagocytosis, granuloma formation, leukocyte recruitment, complement lysis, inflammation, healing responses	Clonal expansion of antigen-specific B and T cells, antigen-specific immunoglobulins
pdigmatic kidney diseases	Urinary tract infection, acute kidney injury, glomerulosclerosis, tubulointerstitial fibrosis, crystal nephropathies C3 glomerulopathy	IC glomerulonephritis, allograft rejection, interstitial nephritis

*IC* immune complex.

Most kidney diseases involve inflammation. Adaptive immunity predominates in kidney disorders that are related to foreign antigens (e.g. in postinfectious glomerulonephritis) or autoantigens (Table 1). Autoantigens may come from within the kidney (e.g. in anti-glomerular basemement membrane glomerulonephritis or renal transplantation) or from extrarenal sources (e.g. in IgA nephropathy). As such immune complex-related glomerulonephritis, allograft rejection or antigen-mediated interstitial nephritis are pdigmatic disorders in which systemic immunosuppression can suppress the immune responses that are regulated outside the kidney.

Innate immunity is the predominant immune response in antigen-independent types of inflammation, such as toxic, ischemic, or traumatic kidney injury, which often present as acute kidney injury where the inflammatory component largely determines renal immunopathology and dysfunction [14]. For example, experimental interventions that suppress inflammation in acute kidney injury, e.g. by blocking pro-inflammatory cytokines and chemokines or by ablating pro-inflammatory leukocyte subsets, largely abrogates tubular cell necrosis and the clinical syndrome of acute renal failure [15]. Tubular necrosis exposes danger signals from dying tubular cells or the tubular lumen to Toll-like receptors or the NLRP3 inflammasome in renal dendritic cells, which sets-off the inflammatory response [16-19]. Also crystal-induced renal inflammation and kidney injury largely depend on NLRP3 inflammasome-mediated induction of interleukin-1β secretion by renal dendritic cells [20]. Innate immunity also drives C3 glomerulopathy where glomerular complement activation is independent of immune complex disease [21]. Innate immunity orchestrates immediate host defense during infective pyelonephritis with uropathogenic bacteria, which may drive renal abscess formation as a form of collateral tissue damage [22,23]. Neutrophil recruitment and neutrophil-mediated immunopathology is a major element of renal immunopathology in renal infection but also in acute tubular necrosis or renal vasculitis, in which innate immunity plays a major role. Finally, even in those diseases that are not directly triggered by immune mechanisms, innate immunity is at least involved in that inflammation that comes with tissue remodeling. Macrophage infiltrates do not always necessarily contribute to renal injury but also to wound healing [24] as macrophage depletion in the healing phase of kidney injury delays kidney regeneration [25-27]. In addition, the role of such wound-healing macrophage phenotypes in driving kidney fibrosis is well established [28]. As such innate immunity is involved in tissue remodeling of all chronic and progressive kidney diseases even such as diabetic nephropathy, Alport nephropathy or polycystic kidney disease [29-32].

But where is the field going? The complex cross-talk between innate and adaptive immunity remains a challenge also for the future [33]. For example, ischemia-reperfusion injury is a trigger of renal allograft rejection but how does that work mechanistically [34,35]? How can monocytes confer allorecognition [36]? What is the role of the recently described innate lymphocytes in kidney disease [37]? How do γδT cells, NKT cells, and B1 cells link innate and adaptive immunity in kidney disease? Can the immunosuppressive potential of regulatory T cells be used for therapeutic purposes? What are the innate and what are the adaptive immune functions of the spectrum of the mononuclear phagocyte phenotypes inside the kidney [38]? And finally, when will we finally implement the novel immunoregulatory drugs that are so successful in other medical disciplines also into treatments for patients with kidney diseases? These and other exciting questions are awaiting to be addressed by nephro-immunologists at the bench and immuno-nephrologists at bedside.

## Competing interests

The author declares that he has no competing interests.

## Pre-publication history

The pre-publication history for this paper can be accessed here:

http://www.biomedcentral.com/1471-2369/14/138/prepub
